# Osmolytes ameliorate the effects of stress in the absence of the heat shock protein *Hsp104* in *Saccharomyces cerevisiae*

**DOI:** 10.1371/journal.pone.0222723

**Published:** 2019-09-19

**Authors:** Arnab Bandyopadhyay, Indrani Bose, Krishnananda Chattopadhyay

**Affiliations:** 1 Structural Biology & Bio-Informatics Division, CSIR-Indian Institute of Chemical Biology, Kolkata, India; 2 Department of Biology, Western Carolina University, Cullowhee, North Carolina, United States of America; North-Eastern Hill University, INDIA

## Abstract

Aggregation of the prion protein has strong implications in the human prion disease. Sup35p is a yeast prion, and has been used as a model protein to study the disease mechanism. We have studied the pattern of Sup35p aggregation inside live yeast cells under stress, by using confocal microscopy, fluorescence activated cell sorting and western blotting. Heat shock proteins are a family of proteins that are produced by yeast cells in response to exposure to stressful conditions. Many of the proteins behave as chaperones to combat stress-induced protein misfolding and aggregation. In spite of this, yeast also produce small molecules called osmolytes during stress. In our work, we tried to find the reason as to why yeast produce osmolytes and showed that the osmolytes are paramount to ameliorate the long-term effects of lethal stress in Saccharomyces cerevisiae, either in the presence or absence of *Hsp104*p.

## Introduction

Protein misfolding and its aggregation has been implicated in a number of neurodegenerative diseases [1]. For example, alpha synuclein, an intrinsically disordered protein, aggregates forming Lewy bodies in the pathophysiology of Parkinson’s diseases [2]. Likewise, aggregation of Superoxide Dismutase (SOD1) is believed to be responsible for Amyotrophic Lateral Sclerosis (ALS) [3]. While many of these diseases, e.g. Alzheimer’s diseases, Parkinson’s diseases and ALS are not found to be infectious, Prion disease or transmissible spongiform encephalopathy is an infectious amyloid disease [4]. Human forms of prion diseases include Creutzfeldt-Jakob disease, Gerstmann-straussler-scheinker disease and fatal familial insomnia [5,6,7]. The most common animal form of Prion disease is bovine spongiform encephalopathy or ‘mad cow’ disease, which is transmissible to humans [8]. It has been hypothesized that a conformational change in prion protein (PrP^C^) into another form (PrP^SC^) makes the protein amyloidogenic, which then aggregates leading to the propagation of the disease [9].

A tightly controlled cellular quality control mechanism regulates protein folding and aggregation inside cellular environment [10]. There are several important components which play crucial role in the maintenance of cellular proteostasis. One of these components are the molecular chaperone proteins, which facilitate stabilization of the natively folded active proteins by reducing aggregation; although their mechanism of action is widely debated [11]. In addition to the protein-based chaperones and heat shock proteins, naturally occurring small molecules have been shown to protect and preserve the native structure of a protein in cellular environments. Many of these small molecules are osmolytes, which include amino acids, polyols and sugar derivatives [12]. These are often referred as the molecular chaperones of the cell. Although the molecular mechanism for osmolyte-induced protein stability has been extensively studied by Street et al, it is not understood how the protein-based and small molecule components of the cellular quality control mechanism influence each other to regulate protein homeostasis [13].

In this paper, we have studied the roles of a heat shock protein, *hsp104* and a small molecule chaperone, trehalose [14], on the in vivo folding and aggregation of the prion protein, Sup35, using yeast as a model system [15]. There are several prion-like proteins, which exist in different fungi including yeast [16]. These proteins are not infectious to humans but carry the same transmissible phenotype as human prion protein. With human prions, an infectious disease propagates through a self-sustaining modification in the structure of a normal protein within the cell, seemingly without the help of any nucleic acid [17]. With yeast prions, a similar mechanism yields a new heritable metabolic state, seemingly without a change in any nucleic acid. The yeast Sup35 protein contains a human prion-like domain [18]. A conformation changes similar to human prion protein between the PrP^C^ and PrP^SC^ form has been found also with Sup35 and the proposed mechanism of the propagation of Sup35 aggregation has been found to be identical to that of mammalian prion protein. Sup35 has been used extensively as a model to study the prion disease in yeast [18].

We have studied the aggregation of Sup35 inside live yeast cells using overexpressed GFP-tagged Sup35 in *Saccharomyces cerevisiae*. Overexpression of Sup35 in yeast has been shown to induce its prion properties. A physical stress in the form of heat shock and a guanidium hydrochloride induced chemical perturbation have been applied to the yeast cells. Using a *hsp104* knockout strain of yeast, we have shown that the contribution of trehalose is comparatively more crucial than *hsp104* function for the protection of Sup35 *in vivo*, particularly during conditions of high stress.

## Materials and methods

### Materials

All osmolytes (sucrose, mannose, sorbitol, mannitol, trimethylamine N-oxide, trehalose), amino acids, lithium acetate, Tris-HCl, EDTA and salmon-sperm DNA (ssDNA) were purchased from Sigma-Aldrich (St. Louis, MO). Yeast extract, dextrose, peptone, agarose and polyethylene glycol (PEG) were purchased from Hi-media Laboratories (Mumbai, India).

### Yeast strains, plasmid and growth media

The wildtype (WT) *S*. *cerevisiae* strain (*MATa his3-Δ200 leu2-Δ1 lys2-801 trp1-Δ63 ura3-52*), a kind gift from Daniel Lew, was used in all experiments [19]. The haploid *hsp104* knockout strain, the pRSCUP-*SUP35-GFP* plasmid (Addgene#1087) and pJCSUP35(1–253) plasmid (Addgene#1089) were obtained from Addgene. Yeast strains were maintained on YPD (Yeast-Extract Peptone Dextrose) media (10gm Yeast extract, 20gm Peptone, and 20gm Dextrose per litre) with or without agarose at 30°C, unless otherwise noted. Yeast cells harbouring the plasmid were selected and maintained on synthetic dextrose medium (0.67gm yeast nitrogen base, 2 gm dextrose, 2gm bactoagar), lacking the amino acid uracil (SDM-ura) as mentioned by Abelson with some modifications [20]. Expression of the Sup35-GFP protein was induced by adding 25μM copper sulphate to cells at OD600 of 0.6 in SDM-ura media.

### Purification of Sup35 protein

pJCSUP35(1–253) plasmid was transformed into E.coli (BL21 DE3 strain) [21,22]. Recombinant Sup35 was over-expressed in E.coli (BL21 DE3 strain). The overexpression of Sup35 was induced using 1 M IPTG. Followed by induction, the cells were allowed to grow for 3.5 hours. The cells were pelleted down by centrifuging at 6000 rpm for 15 minutes at of 4 degrees which was followed by resuspension in pre-chilled lysis buffer (20 mM Tris-HCl + 500 mM NaCl, pH 8.0). After thorough re-suspension in lysis buffer the cells were subjected to sonication (20 pulses, each for 30 seconds pulse time and an interim time frame of 1 minute). Unbroken cells and debris were removed by another act of centrifugation at 10,000 rpm for 10 minutes. The soluble fraction obtained thereafter was carefully removed and allowed to bind to Ni-NTA agarose resin. The Ni-NTA column was washed with 40 ml wash buffer (20 mM Tris-HCl, 500 mM NaCl and 50 mM imidazole, pH 8.0) followed by elution with 20 mM Tris HCl, 500 mM NaCl and 500 mM imidazole, pH 8.0. The eluted fractions were pulled according to their tentative protein content as per their absorbance at 280 nm. The post elution fractions were subjected to dialysis in 20 mM Na-phosphate buffer pH 7.5. In all our protein concentration measurements UV-VIS was deployed and Sup35 concentration was determined by considering the monomeric molar extinction coefficient of 29,000 M−1 cm−1 at 280 nm. The identity of the protein was confirmed by SDS PAGE.

### Yeast transformation

Yeast cells were transformed as per Gietz and Woods with some modifications [23]. Briefly, yeast cells were cultured in YPD liquid broth overnight at 30°C in a shaking incubator, diluted to an OD600 of 0.25 in fresh media and allowed to grow to an OD600 of 0.6. The cells were pelleted by centrifugation at 1000g at room temperature for 10 minutes, washed twice with double-distilled water and then with 10ml LTE buffer (100mM Lithium Acetate, 10mM Tris-HCl, 1mM EDTA, pH 7.4), before being resuspended in 500μl of the same buffer. 10ng boiled ssDNA solution in 300μl transformation mix (40% PEG 3350 in LTE buffer, pH 7.4) and 5ng of plasmid DNA were added to these yeast cells. After incubation at 30°C for 30 minutes, the cell suspension was heat-shocked at 42°C for 15minutes, and then incubated on ice for 15minutes. 100μl of this mixture was plated on SDM-ura media and incubated at 30°C for 2–3 days.

### FACS analysis

Cells grown in YPD (Yeast-Extract Peptone Dextrose) or SDM-ura media with CuSO_4_ were diluted to 10^5^cells/ml for performing FACS analysis [24], on a BD LSRFortessa™ cell analyser flow cytometer (BD Biosciences). Cells were excited at 488nm.

### Confocal microscopy and image analysis

Yeast cells were grown to an OD600 of 1 and then heat stressed by placing them in a water bath at 46°C for 30minutes, or chemically stressed with 5mM guanidium hydrochloride for 24 hours, or left unstressed. Agarose beds of SDM-ura media, containing various osmolytes, with or without copper sulphate, were prepared on glass slides. Yeast cells were added to the agarose beds and incubated at 30°C for 24 hours, unless otherwise noted, before microscopy.

Confocal microscopy was performed using the Zeiss LSM 510 confocal microscope at 63x magnification and 488nm laser excitation. Imaging speed was set at 5, the pinhole was kept at 1 and 4 averages were taken per field. ZEN2009 and the OriginLab 9.0 software were used for imaging the yeast cells and for graphing respectively. Fields with an average of 200 yeast cells 63x objective were imaged and analysed.

### Western Blot analysis and antibodies

Protein concentrations were measured using BioRad Dc Protein Reagent. Proteins were separated on 4–15% gradient SDS-polyacrylamide gels (BioRad) under reducing conditions and then transferred to polyvinylidene fluoride membrane (Millipore) in Tris-glycine buffer pH 7.5 containing 10% methanol. Filters were blocked at room temperature (RT) in 5% non-fat dry milk in TBST [0.1 M TrisHCl pH 7.5, 0.15 M NaCl, and 0.1% Tween-20 (Sigma)], incubated overnight with primary antibodies at 4°C, washed in TBST (4×5 min) and appropriate secondary HRP-conjugated antibodies were applied for 90 min at RT. Filters were washed as above, developed with ECL (Pierce) and exposed to High lot CL autoradiography film. Films were scanned using an Epson V600 scanner.

Antibodies used (dilutions made in 5% milk): Rabbit polyclonal to GFP—ChIP Grade (Ab290), 1:1000 dilution; Rabbit polyclonal to beta Tubulin (Ab15568), 1:1000 dilution; Rabbit polyclonal to *hsp104p* (Ab2924), 1:1000 dilution; Goat Anti-Rabbit IgG H&L (Alkaline Phosphatase) (Ab6722), 1:1000 dilution and Goat Anti-Rabbit IgG H&L (HRP) (ab205718), 1:1000 dilution.

## ThT fluorescence assay

The protein Sup35 was subjected to mechanical agitation of 200 rpm at 37°C for 7 days. The protein concentrations for the aggregate preparation were kept 30 μM in 20 mM sodium phosphate buffer at pH 7.5. Aliquots were thereafter subjected to 10°M ThT and fluorescence measurements were taken using an integration time of 0.3 s. The steady state fluorescence was monitored using an excitation wavelength of 450 nm, and the values of emission intensity at 485 nm were recorded.

## Circular dichroism

Far-UV CD spectra of Sup35 were recorded using a JASCO J720 spectropolarimeter (Japan Spectroscopic Ltd.). Far-UV CD measurements (between 200 and 250 nm) were performed using a cuvette of 1 mm path length. Protein concentrations of 10°M was used for CD measurements. The scan speed was 50 nm min^−1^, with a response time of 2 s. The bandwidth was set at 1 nm. Three to five CD spectra were recorded in the continuous mode and averaged.

## Results and discussion

### Sup35-GFP protein shows some aggregation when overexpressed

**Fig 1:**
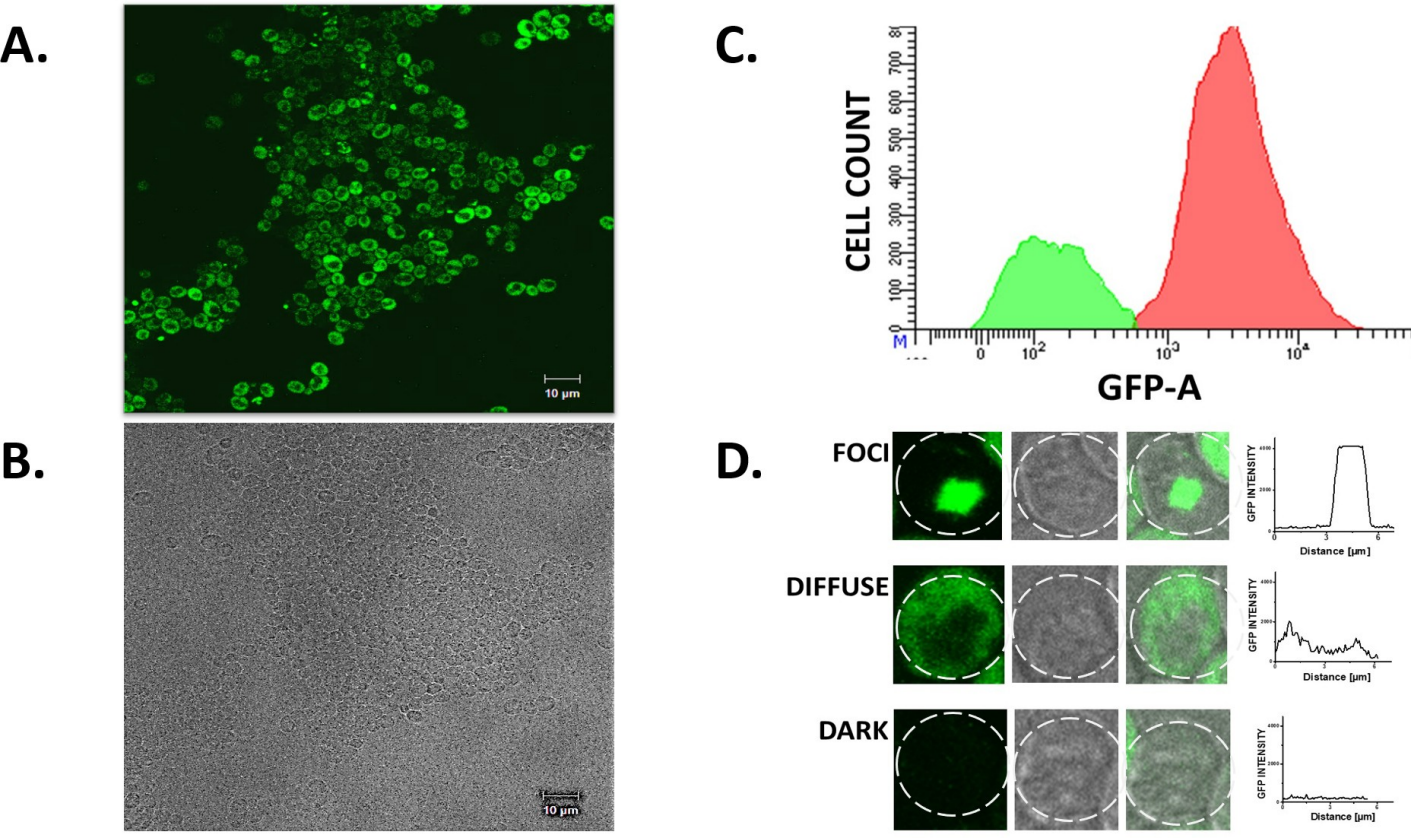
Characterization of wild type yeast cells expressing Sup35-GFP. **(A)** Wild type yeast cells expressing Sup35-GFP. **(B)** DIC image of (A). **(C)** Flow cytometry showing the percentage of fluorescent vs. dark cells in the population. 25% of the cells are dark. **(D)** Types of aggregation of Sup35-GFP in yeast cells. Three different aggregation phenotypes were observed: foci or aggregate (showing a fluorescent foci), diffuse (fluorescence observed throughout the cytoplasm), and dark (no fluorescence). GFP intensity versus distance plots show characteristic patterns for aggregate, diffuse, and dark cells.

**Fig 2:**
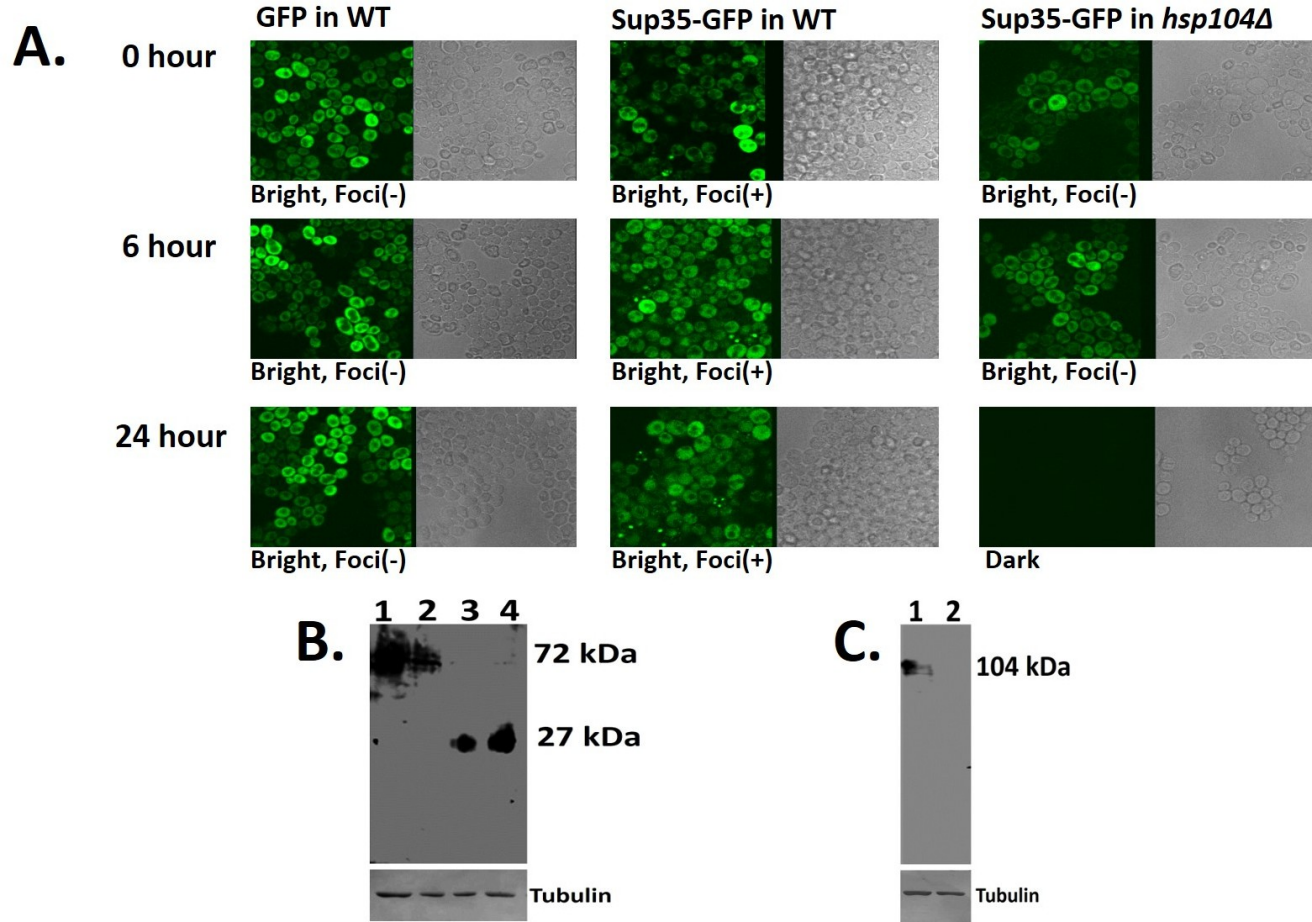
*Hsp104*p function is required for maintenance of Sup35-GFP folding. **(A)** GFP expression in wild type yeast cells at 0, 6, and 24h post-plating. No fluorescent foci (aggregates) were observed in any of the cells. Sup35-GFP expression in wild type yeast cells at 0, 6, and 24 hours post-plating shows the presence of foci. Sup35-GFP expression in an *hsp104∆* strain is not maintained 24 hours after plating. **(B) Expression of Sup35-GFP**. Anti-GFP blot showing the expression of Sup35-GFP in wild type and *hsp104∆* yeast cells after 24 hours incubation, (Lane 1: Sup35-GFP, Lane 2: *hsp104∆* Sup35-GFP, Lane 3: GFP, Lane 4: *hsp104∆* GFP). **(C) Expression of *Hsp104*p**. Anti-*Hsp104*p blot testing the presence of Hsp104p in wild type and *hsp104∆* cells (Lane 1: wild type, Lane 2: *hsp104∆*).

Yeast cells expressing Sup35-GFP protein were visualized by confocal microscopy. Sup35-GFP protein expression was detected in 75% of cells in a wild type yeast culture incubated for 24h at 30°C under normal conditions (Fig 1A, 1B and 1C). Of the cells expressing the fusion protein, 86.5% had a diffuse fluorescence, while the remaining 13.5% showed one or two fluorescent foci (aggregates). It may be noted that GFP tagged Sup-35 has been extensively used to monitor Sup-35 aggregation studies in live yeast cells [24,25,26], and GFP fusion does not change the monomeric nature of the protein. GFP alone did not form any aggregates under our experimental conditions (Fig 2A). The pattern and intensity of fluorescence in diffuse vs. aggregated Sup35-GFP could be differentiated by analysing the volume of the cell to which the protein had localized. The fluorescent foci in the Sup35-GFP aggregates had higher fluorescence over a smaller cellular radius whereas in cells with diffuse expression the fluorescence intensity was lower and present throughout the cytoplasm, as shown in Fig 1D. This suggests that when overexpressed, the Sup35-GFP protein is able to form spontaneous aggregates in a small sub-population of yeast cells even under normal conditions. About 25% of cells in the culture did not show any detectable Sup35-GFP expression.

### Maintenance of Sup35-GFP folding and formation of aggregates requires *HSP104* function

In order to determine the kinetics of aggregate formation in wild type yeast cells, Sup35-GFP protein expression was assayed for pattern and intensity of GFP fluorescence at different time points. As shown in Fig 2A, Sup35-GFP expression and folding is maintained over time in wild type yeast cells and aggregate formation increased from 0 to 24h incubation, as observed through confocal microscopy. The formation of aggregates was not seen in cells expressing GFP alone, confirming that Sup35p has self-aggregation properties, as previously reported.

The heat shock protein, *Hsp104*p, is known to function as a chaperone that is required for protein folding. We wanted to investigate whether *Hsp104*p played a role in initiating and/or maintaining Sup35-GFP folding in yeast. To do so, we analysed Sup35-GFP fluorescence in an *hsp104∆* (*hsp104* knockout) *strain*. Sup35-GFP fluorescence is detectable for the first few hours in this strain (Fig 2A), showing that *Hsp104*p function is not required for the initial expression and folding of the Sup35-GFP protein. However, this fluorescence is absent in cells after 24 hours of incubation, suggesting that *Hsp104*p is required for long term maintenance of proper Sup35-GFP protein folding. It has been shown before that while Hsp104 is needed to maintain aggregation, it does not necessarily initiate the process [27,28,29]. The present data shows that *Hsp104*p is also required for the self-aggregation properties of Sup35-GFP protein since fluorescent protein foci were absent in the earlier time points, in contrast to that found in wild type yeast cells (Fig 2A).

### *Hsp104*p function does not affect Sup35-GFP protein expression

In order to determine whether the lack of *Hsp104*p function affected Sup35-GFP expression, western blots were performed on wild-type and *hsp104∆* strains expressing Sup35-GFP. As seen in Fig 2B, the Sup35-GFP protein is present as a 72kD band in both the wild type and in *hsp104∆* yeast cells, confirming that the lack of Sup35-GFP fluorescence seen in Fig 2A is due to the misfolding of the protein in the absence of *Hsp104*p function and not due to an absence of the protein itself. Fig 2C confirms the lack of *Hsp104*p expression in the *hsp104∆* strain (Fig 2C, lane 2); *Hsp104*p is present as a 102kD protein in the wild type cells only (Fig 2C, lane 1).

### Long-term stress affects protein folding in wild type yeast cells

**Fig 3:**
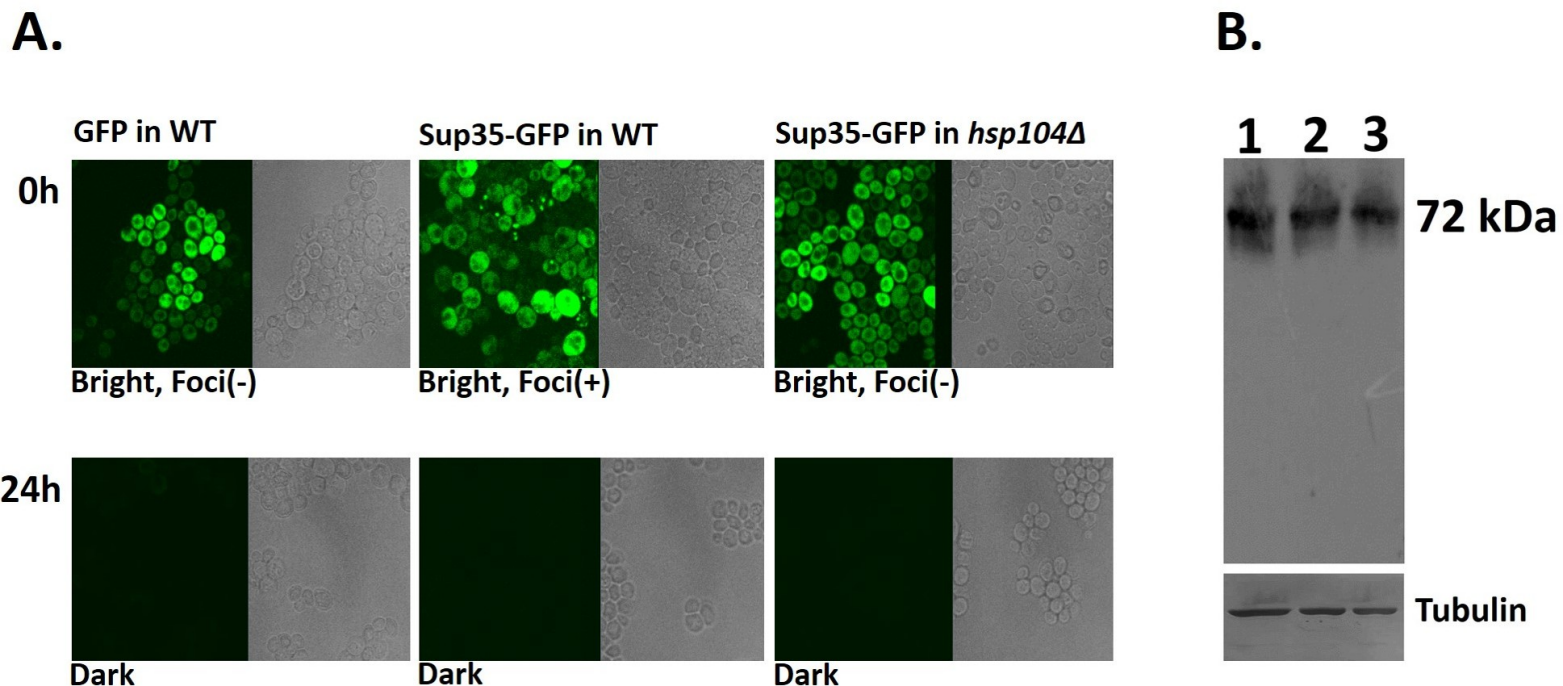
**(A) Effects of stress on Sup35-GFP protein.** Sup35-GFP in wild type and *hsp104Δ* yeast cells when observed under the confocal microscope under no stress (t = 0 hours). Sup35-GFP in wild type and *hsp104Δ* cells after 24 hours incubation in the presence of 5mM guanidium hydrochloride. **(B) Expression of Sup35-GFP under stress conditions.** Sup35-GFP protein in unstressed cells (lane 1), in cells exposed to 5mM guanidium hydrochloride for 24 hours (lane 2), and in cells exposed to 5mM guanidium hydrochloride and 100mM trehalose for 24 hours (lane 3).

Chemical and physical stresses are known to affect protein folding. We investigated the effects of 24 hours exposure to either 5mM guanidium hydrochloride (chemical stress) or a 30minutes exposure to heat shock at 46°C (physical stress), on the folding of the Sup35-GFP protein in wild type and *hsp104Δ* yeast cells. As seen in Fig 3A, both strains showed fluorescent expression of Sup35-GFP at 0 hours and consistent with our previous results, wild type cells showed some protein aggregation which was absent in the *hsp104Δ* cells. There was no significant change in fluorescence pattern or intensity in the two strains after 6 hours incubation in media containing 5mM guanidium hydrochloride, but Sup35-GFP fluorescence was absent in both strains after 24 hours (Fig 3A). This absence of fluorescence was not due to the absence of the protein, as levels of Sup35-GFP were similar in both unstressed and chemically stressed wild type cells (Fig 3B).

The effect of chemical stress on protein folding was not specific to Sup35-GFP, as seen by the absence of fluorescence in wild type yeast cells expressing GFP alone, suggesting that the presence of chaperone function in wild type cells was not able to overcome the effects of long term chemical stress on the unfolding or misfolding of cellular proteins. A similar effect of heat stress on protein folding was also seen in both yeast strains.

### Osmolytes can rescue protein folding in long-term stressed yeast cells lacking *Hsp104*p

**Fig 4:**
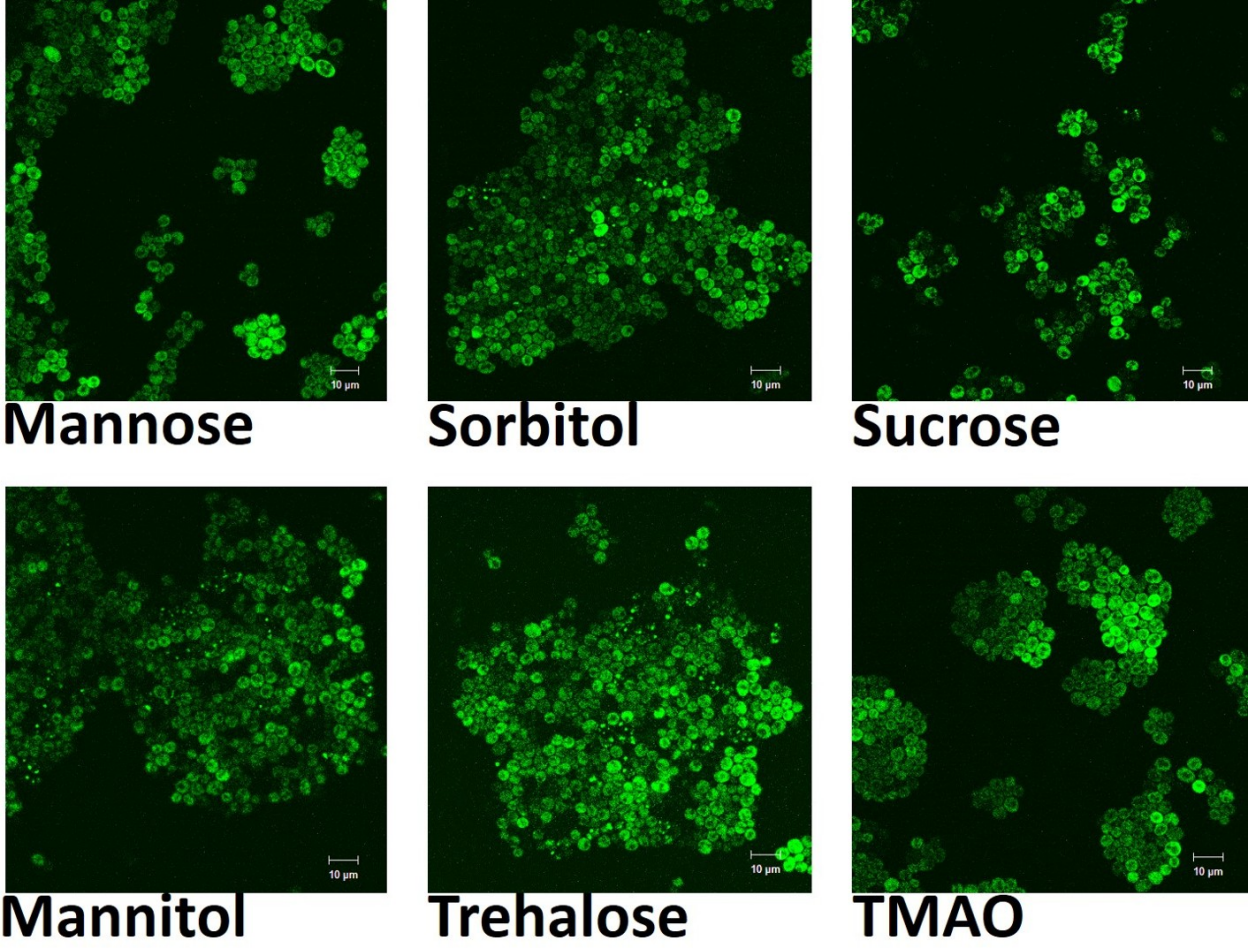
Confocal imaging of yeast cells under stress with different osmolytes. Mannose, mannitol, sucrose, sorbitol, trehalose and TMAO were used as osmolytes in our study. All these osmolytes were able to alleviate the effects of stress on the yeast cells of the wild type strain. The yeast cells had aggregates and they looked bright under the confocal microscope.

Osmolytes are small molecules that are known to stabilize protein folding [13,30,31,32,33]. In order to determine the effects of these small molecules on *in vivo* protein folding, yeast cells were exposed to six different osmolytes in the presence of the stresses described above. The selected osmolytes, namely sucrose, sorbitol, mannose, mannitol, trehalose and TMAO are simple in their composition and are well known to be produced by stressed cells in nature. Cells of almost all organisms accumulate organic osmolytes when exposed to stressful conditions [34]. As shown in Fig 4, wild type cells exposed to physical and chemical stress showed fluorescent Sup35-GFP production when treated with any of these six different osmolytes, suggesting that they are able to revert the yeast cells to the normal unstressed phenotype, including the presence of aggregates in a sub-population. Thus, osmolytes can help maintain proteins in the properly folded conformation during stressful conditions inside yeast cells.

**Fig 5:**
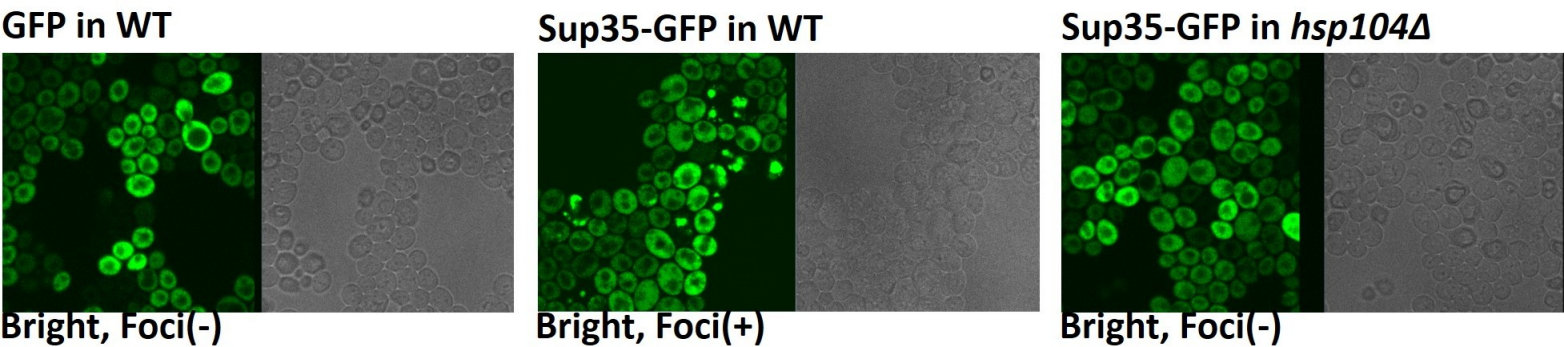
Trehalose stabilize and revive Sup35-GFP protein folding under stress conditions. Sup35-GFP in wild type and *hsp104Δ* cells after 24 hours incubation in 5mM guanidium hydrochloride and 100mM trehalose.

The beneficial effects of these osmolytes on protein folding was evident even in the absence of *Hsp104*p function. Fig 5 shows the effects of 100mM trehalose on Sup35-GFP fluorescence after 24 hours incubation in the presence of 5mM guanidium hydrochloride. Trehalose was able to ameliorate the effects of the chemical stress completely and return protein folding and aggregation to unstressed levels. The other five osmolytes were also able to reverse the effects of stress on the folding of the GFP and Sup35-GFP proteins, both in the wild type (Fig 4) and *hsp104Δ* cells. This suggests that *hsp104*p function is not required for protein folding in the presence of trehalose (and other osmolytes) during long term stress.

To check if aggregation under in vitro condition and the effect of osmolytes are similar inside cells and under in vitro conditions, we repeated few representative experiments in aqueous buffer. To monitor the aggregation in vitro, we used the ThT fluorescence assay. The protein was found to form aggregates as a large increase of ThT fluorescence was observed with a sigmoidal profile (S1 Fig), as shown by others before [35]. In the presence of guanidium hydrochloride (gdnHCL) and gdnHCL with trehalose (with concentrations similar to that used in cells experiments), we observed a large decrease in aggregation (S2A and S2B Fig). A conformational analyses using far UV CD under in vitro condition also remained inconclusive. This is because; the far UV CD profile of Sup35 in aqueous buffer is featureless (S2C Fig), also observed by others before [18], and no significant effect of the osmolyte was observed.

## Conclusion

Over the last 20 years, it has become apparent that the heat shock proteins are key elements overriding the equilibrium between aggregation and maintaining the properly folded conformation of the proteins inside live cells [36,37]. Our data show that a similarly antique and well-preserved mechanism, the production of osmolytes [33,38,39], also contributes critically to this equilibrium. Osmolytes are naturally occurring small molecules with the ability to protect and preserve the native structure of a protein [33,39,40]. They are often referred to as the molecular chaperones of the cell [41]. Trehalose, a sugar which is composed of two molecules of glucose, has been considered to be a natural osmolyte [42]. Das et al. has shown the trehalose induced structural modulation of Bovine Serum Albumin at ambient temperature [39]. They have also shown how trehalose mediates the stabilisation of cellobiase aggregates from the filamentous fungus *Penicillium chrysogenum* [33]. A significant amount of research had shown that trehalose can shield native proteins from a huge variety of stresses in-vitro [29,39,43], but the role of trehalose and other similar osmolytes in stress tolerance in-vivo triggered much controversy. We have shown that externally applied osmolytes successfully overcomes the “long term” effects of both the chemical (gdnHCl) and thermal (heat shock) stresses in-vivo, although, we cannot rule out the possibility that the osmolytes fold Sup35 up indirectly by facilitating the action of other chaperones. These inferences resolve observations that formerly seemed contradictory, and are of significance for stress tolerance as well as the evolution of different osmolyte systems in maintaining the proteins in the properly folded conformations during stress.

Our findings provide a clear picture of the capabilities of heat shock proteins and osmolytes in protecting cells from the quirks of their environment. Proteins inside cells are not maintained in the properly folded conformation due to stress [44,45,46]. Osmolytes functions first against this process, by stabilizing proteins in their native state [47,48]. Proteins are bound by heat shock proteins to maintain them in the properly folded conformation during stress [49,50]. In our studies we found out that, osmolytes alone, were able to protect the *hsp104* knockout yeast cells during severe physical and chemical stress. Here, osmolytes act again, by maintaining the proteins in the properly folded conformation, when the protein repair machinery is overwhelmed. Thus, the relative importance of heat shock proteins and osmolytes, which has been a long-standing subject of controversy, is now clear, although the effect of other related heat shock proteins like Ydj1 or Ssa1 is being investigated in our lab to see whether these effects are part of much larger machinery.

Our present work, together with previous observations [51,52], establishes that osmolytes are crucial in maintaining the proteins in the properly folded conformation during stress. As described by Alexander et al [53], there is a lack of correlation between trehalose accumulation induced by various stresses in *Saccharomyces cerevisiae*. They have also shown that there is not a single and common way for cells to accumulate trehalose in response to various types of stress. The intracellular concentration of trehalose under the various conditions of stress, at several timepoints, by using different types of techniques has already been measured by Tapia et al [54], Gibney et al [55] and Ratnakumar et al [56]. Thus, we assumed similar baseline level of trehalose concentration both for wild type and *hsp104* knockout cells during similar experimental conditions and timepoints [57]. Alternatively, we assumed that *hsp104* knockout cells would not substantially affect the trehalose producing pathways under stress, and hence the observed effect of trehalose is the influence of external addition only.

In our work, we have observed the “long term” effects of chemical and physical stress on yeast cells in the absence and presence of six different osmolytes. Lindquist and other well-known scientific groups had previously showed that gdnHCL treatment for 2–6 hours can cure the [psi+] [58,59,60,61,62,63]. Interestingly, in our study, we found that gdnHCL treatment for a very long time, that is for 24 hours, caused the yeast cells to appear almost dark under the confocal microscope and thus is considered as a type of severe stress for the yeast cells. Darkening of the yeast cells under the confocal microscope proved that the yeast cells were probably experiencing stress, as the proteins were not properly folded. The molecular mechanism for osmolyte-induced protein stability has already been shown by Street et al [13]. They showed that protecting osmolytes push the protein folding reaction equilibrium towards the native state. Our work also shows that osmolytes help to maintain the proteins in their native folded conformation. This is probably because the osmolytes has shielded the yeast cells from the effects of stress by maintaining the proteins in the properly folded conformation. Western blot data has shown that the expression of proteins was similar in both the stressed and unstressed cells. All the six protecting osmolytes were able to help yeast cells to withstand the “long term” effects of both the physical and chemical stress situations by maintaining the proteins in the properly folded conformation. Thus, our work shows that osmolytes are crucial in maintaining the proteins in the properly folded conformations during stress and probably answers the paradox, as to why, in spite of having heat shock proteins, yeast still produce osmolytes during stress. These observations suggest a number of important uses. In the present scenario, an increasing number of human diseases are the result of protein aggregation[64,65]. Many compatible solutes have been proposed for therapeutic use [39,66,67]. One of the direct consequences of a trehalose coated microenvironment has been predicted by Das et al[39]. However, here lies the danger [68]. The design of such strategies must take into account measures to overcome the potential interference of these osmolytes such as those observed in our study. Attempts of developing trehalose based formulations for treating misfolding diseases such as prion disease can only be successful if we can enhance our understanding of the in-vivo mechanisms of action of trehalose and other osmolytes. The evolving understanding of these compounds and their unfamiliar properties opens broad avenues for further investigation and promising applications [29].

## Supporting information

S1 Fig Intrinsic aggregation profile of Sup35 in-vitro using Thioflavin T fluorescence. Aggregation tendency of Sup35 is shown here.
(TIF)

S2 Fig Fluorescence and CD spectra of Sup35 in the presence of gdnHCL and various osmolytes. **(A)** ThT Fluorescence spectra during the aggregation of Sup35 at 0 hours. **(B)** Fluorescence spectra during the aggregation of Sup35 at 113 hours. **(C)** Far UV CD spectra of Sup35 in the presence of gdnHCL and trehalose.
(TIF)
