# Supplementary figures and images for: Osmolytes ameliorate the effects of stress in the absence of the heat shock protein Hsp104 in Saccharomyces cerevisiae
Source: PLoS One. 2019 Sep 19;14(9):e0222723. doi: 10.1371/journal.pone.0222723 (PMC6752772; doi:10.1371/journal.pone.0222723)

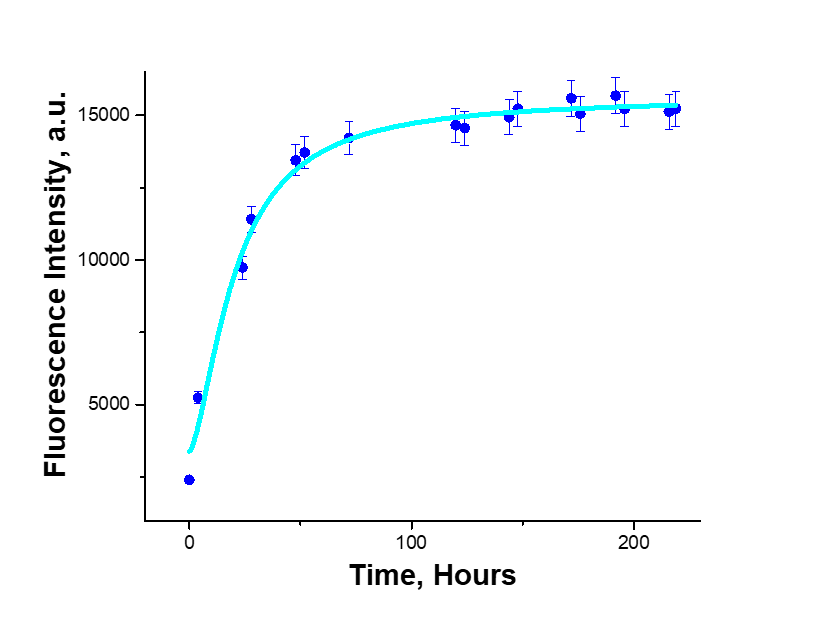

Supplement: S1 Fig — Aggregation tendency of Sup35 is shown here. (TIF) [file pone.0222723.s001.tif]

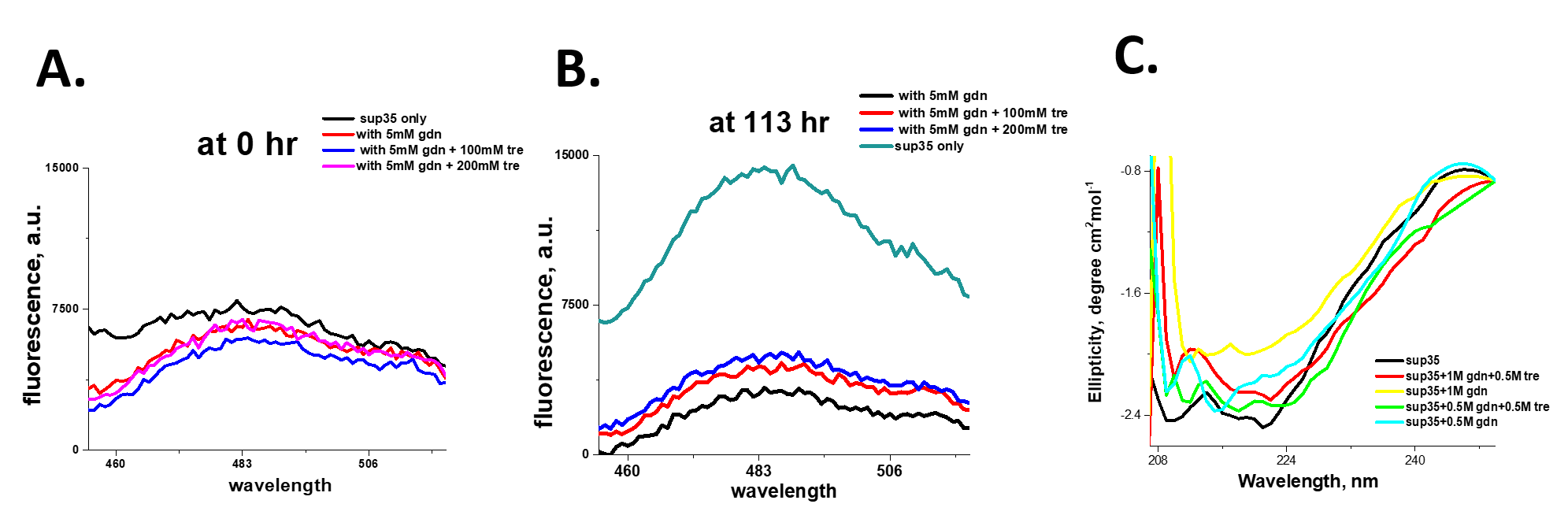

Supplement: S2 Fig — (A) ThT Fluorescence spectra during the aggregation of Sup35 at 0 hours. (B) Fluorescence spectra during the aggregation of Sup35 at 113 hours. (C) Far UV CD spectra of Sup35 in the presence of gdnHCL and trehalose. (TIF) [file pone.0222723.s002.tif]
